# Effect of a 5000 ppm fluoride toothpaste and a 250 ppm fluoride mouth rinse on the demineralisation of dentin surfaces

**DOI:** 10.1186/1756-0500-2-147

**Published:** 2009-07-23

**Authors:** Mozhgan Bizhang, Yong-Hee P Chun, Mai-Trinh Winterfeld, Markus J Altenburger, Wolfgang HM Raab, Stefan Zimmer

**Affiliations:** 1 Heinrich-Heine-University, Dept. of Operative and Preventive Dentistry and Endodontics, Düsseldorf, Germany; 2 Biologic and Materials Sciences, Periodontics and Oral Medicine, University of Michigan, Ann Arbor, MI, USA; 3 Dept. of Operative Dentistry and Periodontology, Albert-Ludwig University, Freiburg, Germany; 4 Department of operative Dentistry, university Witten/Herdecke, Witten, Germany

## Abstract

**Background:**

The purpose of this study was to test the null hypothesis that there is no difference between the effect of (1) a 5000 ppm fluoride toothpaste, and (2) a 250 ppm fluoride mouth rinse on demineralized human dentin surfaces, against the alternative hypothesis of a difference.

**Findings:**

Dentin specimens were obtained from the cervical regions of 45 extracted human third molars. Half the surface of each specimen was sealed with a self-etching adhesive system and served as the reference surface. The dentin specimens were randomly assigned to one of the three groups, 5000 ppm fluoride toothpaste (Duraphat), 250 ppm fluoride mouth rinse (Meridol) and distilled water (negative control).

An intraoral appliance was made for one volunteer. In each test cycle, 15 specimens were inserted in the appliance and worn for 24 hours a day, over a period of three weeks.

Once daily, the appliance was immersed in the agent being tested; either toothpaste slurry, mouth rinse or distilled water for 60 seconds.

Demineralization was assessed in terms of lesion depth (μm) and mineral loss (vol. % × μm) by transversal microradiography. Data analysis was accomplished using Kolmogorov-Smirnov test and ANOVA (SPSS 12.0).

Statistically significant differences for mineral loss and lesion depth were found between the toothpaste and the mouth rinse as well as between the toothpaste and the control group, but not between the mouth rinse and the control group.

**Conclusion:**

Within the limitations of this study, the results suggest that treatment of demineralised dentin with a toothpaste containing 5000 ppm fluoride may considerably reduce mineral loss and lesion depth on exposed dentin.

## Background

Fluoride has been a vital agent in caries prevention since the last century. The caries preventive effect of fluoride is mainly attributed to its effects on demineralization/remineralization of dental hard tissue at the tooth-oral fluids interface. The substitution of hydroxyl ions by fluoride in the mineral crystal, allows for a tighter packing of calcium hydroxyapatite thereby decreasing the solubility of the mineral. In an acidic environment, for example, following sugar intake, fluorohydroxyapatite is more resistant to dissolution than hydroxyapatite. At the apatite-solution interface hydroxyl ions are replaced by fluoride ions resulting in remineralization. [1] Therefore, the loss of mineral is prevented before it can be detected microscopically.

Fluoride plays an important role in the control of root caries [1], by not only reducing the caries progression rate but also by inducing the arrest of active lesions [2]. Topically applied fluoride, in different concentrations, has proven to be capable of reducing root caries development in vitro [3], in situ [4] and in vivo [5].

The function of collagen in the remineralization of dentinal lesions is controversially discussed. Early studies found that collagen did not seem to contribute either positively or negatively to embedding fluoride ions into the hydroxyapatite structure [1,6]. More recently, collagen has been shown to be critical for dentin remineralization [7], in the presence of fluoride. Various clinical studies have shown that the introduction of fluoride shifts the balance from demineralization to remineralization or lesion arrest [8]. Furthermore, the cariostatic property of a dentifrice containing both amine fluoride and stannous fluoride (ASF) (250 ppm fluoride) has been documented [9].

The rapid progression of root caries may be limited by a high fluoride concentration. In enamel, increasing the concentration of fluoride leads to a reduced loss of calcium [10]. Therefore, in an effort to prevent root caries, a toothpaste containing 5000 ppm of sodium fluoride (NaF) is tested in this study. An intra-oral experimental caries model[11,12] is exploited here, for the evaluation of the anti-caries effect of the high fluoride concentration toothpaste. The null hypothesis tested was: there is no difference in the effect of fluoride, in different concentrations, on the demineralization of human dentin surface, against the alternative hypothesis of a difference.

## Materials and methods

### Experimental Design

The study was approved by the ethical committee of the University of Duesseldorf, Germany (No 3256). Informed consent was obtained from the subject. One volunteer, with no signs of active caries or periodontal disease, but with moderate previous caries experience (FS = 20) participated in this study. The subject was in good general health and had not taken antibiotics for at least one month. The subject was instructed to use a non-fluoridated toothpaste (Aronal; GABA, Basel, Switzerland), starting four weeks prior to and continuing throughout the experimental period.

**Figure 1:**
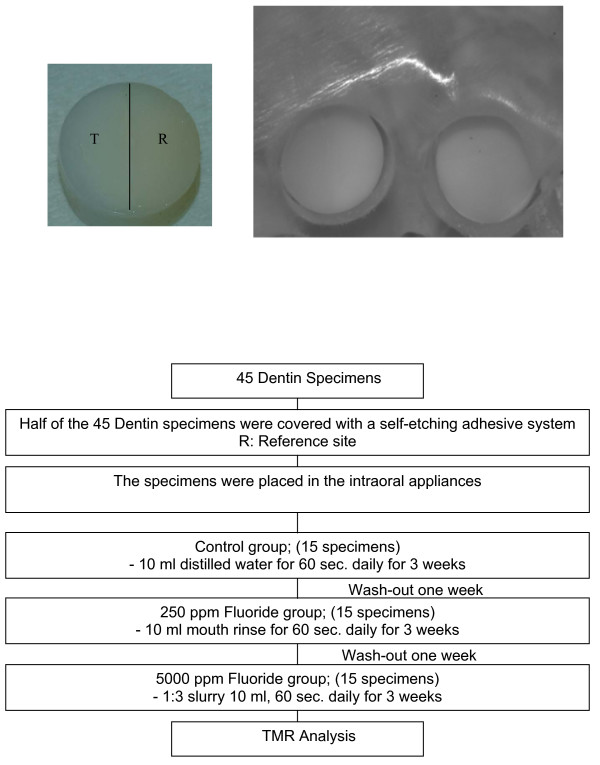
**Appliance design and flow chart of the procedures of the experimental design**. The dentin specimen with test and reference sites, T: test site; R: Reference site (left) and the intraoral appliance with the incorporated dentin specimen adapted to the cast of a volunteer. The dentin specimen inserted into the mandibular appliance (right).

Specimens from forty-five extracted, caries-free, human third molars were used for the intraoral demineralization model. Dentin specimens were obtained from the cervical region, on either the buccal or lingual surface of the crown, using a trephine bur of 6 mm diameter. The enamel layer was entirely removed from this hard tissue cylinder. This was confirmed by inspection of the specimens under a dissecting microscope (8 × magnifications). The specimens were sterilized by radiation. After sterilization, all specimens were cleaned with a soft toothbrush under tap water and air dried. The intraoral mandibular appliance with space for fifteen specimens was fabricated. The specimens were positioned in the appliance and fixed with wax (Fig. 1). In order to promote plaque accumulation, the specimens were mounted leaving a space of 1-mm between the upper level of the appliance and the surface of the specimen.

The agents being tested in this study were 5000 ppm sodium fluoride (Duraphat, Colgate-Palmolive, Piscataway, New Jersey, USA) and 250 ppm amine fluoride and stannous fluoride (Meridol, GABA, Lörrach, Germany). Specimen without fluoride treatment served as controls. In the test groups, an internal reference was required for the subsequent analysis of the effect of the fluoride containing agents. Therefore, half of the dentin disk was sealed with a self-etching adhesive system to protect it from demineralization.

Prior to the study, the subject received an oral prophylaxis at the initial visit to establish a plaque-free and calculus-free baseline. In order to obtain the greatest possible plaque accumulation, the appliance was worn 24 hrs a day over the three-week experimental period. At meal times and during brushing, the appliance was stored in a 10% sucrose solution to promote plaque accumulation. To account for home-care measures, instead of brushing the appliance with toothpaste, it was immersed in either toothpaste (5000 ppm sodium fluoride) slurry (1:3 w/v toothpaste/water), 10 ml mouth rinse or 10 ml distilled water, according to the experimental group, for 60 seconds once daily.

In the control group, the dentin disk remained untreated. This allowed unrestricted exposure to the intraoral environment and therefore facilitated demineralization of the surface. At the end of the period of 3 weeks, the volunteer had a one week recess; after which the next set of specimens were fixed in the appliance and exposed to the oral microflora. Specimens were removed from the appliance and stored in Ringer's solution (0.9%, Deltaselect, Deltaselect GmbH, Pfullingen, Germany) before they were analyzed. The Ringer's solution was changed daily in order to prevent bacterial and fungal contamination.

### Microradiography

For mineral analysis and lesion depth, the specimens were sectioned to a thickness of 100 μm and processed for transversal microradiography as described by Kielbassa et al [13].

The mineral loss (Delta Z) was calculated as the difference in mineral content (Vol. % × μm) of the dentin in the reference site and that of the experimental site over the depth of lesion. Lesion depth was defined as the distance from the surface (0%) to the location in the lesion where the mineral content was less than 95% of the mineral content in sound dentin. For each section, the experimental area and the reference area were measured at the centre of each section.

### Statistical Methods

All statistical analyses were performed with SPSS, version 12.0 for Windows. Normality of the data of each group was analyzed using the Kolmogorov-Smirnov test. The data analysis was accomplished using one-way ANOVA plus LSD (Least Significant Difference) testing for mineral loss and lesion depth to compare the three groups. The tests were performed with α = 0.05 to test for significant differences between the three groups.

## Results

Demineralization was reflected by mineral loss and lesion depth as measured by microradiography. The data of the lesion depth and mineral loss were normally distributed. (Table 1)

**Table 1:** Mineral loss (Vol. % × μm) and lesion depth (μm) of dentin.

Methods	n	Mineral loss (Vol.% × μm)			Lesion depth (μm)		
		
		Range	Mean	SD	Range	Mean	SD
Control	15	-79.00–483.90	328.56 ^a^	137.25	6.70–67.30	19.00 ^a^	14.24
250 ppm F	15	151.50–258.10	218.64 ^b^	28.40	-14.40–68.70	20.80 ^b^	18.10
5000 ppm F	15	-150.80–167.90	60.70 ^a, b^	76.35	-29.30–20.10	5.32 ^a, b^	11.56

The dentin specimens were exposed for 3 weeks to demineralization and treated with fluoride.

Values with the same superscript (^a, b^) within columns revealed a statistically significant difference (p < 0.05).

SD = standard deviation; F = fluoride

**Figure 2:**
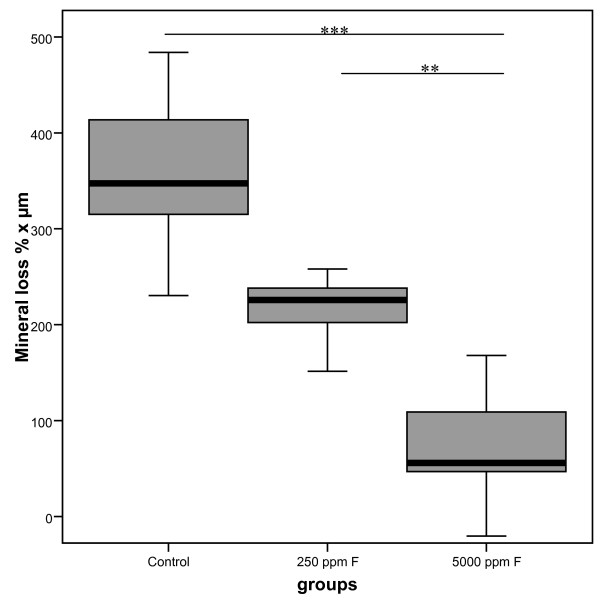
**Mineral loss (Vol. % × μm) in dentin treated with fluoride (n = 15)**. The mean mineral loss from dentin was significantly lower in the group treated with 5000 ppm fluoride compared to the 250 ppm fluoride group and the control group. Mineral loss was not statistically significantly different between the 250 ppm fluoride and control group. (**p < 0.01, ***p < 0.001).

Data reflecting the mineral volume percentage distributions in each of the three groups is presented. The mean mineral loss from dentin was significantly lower in the group treated with 5000 ppm fluoride compared to the 250 ppm fluoride group and the control group (Fig. 2). Mineral loss was not found to be statistically significantly different between the 250 ppm fluoride and control groups

**Figure 3:**
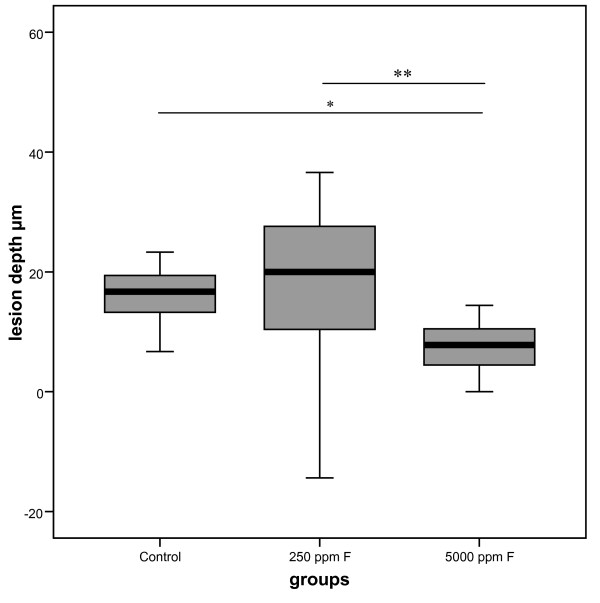
**Lesion depth (μm) in dentin treated with fluoride (n = 15)**. The lesion depth in dentin of the 5000 ppm fluoride group was significantly lesser than in the 250 ppm fluoride group and the control group. In addition, the lesion depth of the 250 ppm fluoride group and the control group were not statistically significantly different. (*p < 0.05, **p < 0.01).

The lesion depth in dentin of the 5000 ppm fluoride group was significantly lesser than in the 250 ppm fluoride group and the control group. The lesion depth of the 250 ppm fluoride group and the control group were not found to be statistically significantly different (Fig. 3).

## Discussion

The Intraoral demineralization model, as employed in this study has often been used to evaluate the cariogenic and anti-cariogenic properties of fluoride containing dental products [14]. Such an experiment may be considered as an intermediate stage between an in situ and an in vitro study. In situ studies also allow a satisfactory control of clinical conditions related to the development of caries [11,15]. From a biological perspective, it is virtually impossible for in vitro models to adequately simulate the complex and diverse intra-oral conditions contributing to caries development. Nevertheless, one approach is the *in vitro *"artificial mouth" system, which comprises of bacterial plaque and artificial saliva. Unlike other clinically based trials examining the effects of preventive agents on intraoral caries development, the intra-oral demineralization model offers the advantage of accurate measurement of changes in lesion size and mineral content, while simultaneously enabling the analysis of treated and non-treated sites of the same dental tissue [16]. The null hypothesis tested in this study was that there is no difference in the effect of fluoride, in different concentrations, on the demineralization of human dentin surface. In the present study, a statistically significant difference was observed between the 5000 ppm fluoride toothpaste and the 250 ppm fluoride mouth rinse in terms of the lesion depth and mineral loss. Mechanistically, fluoride replaces hydroxyl ions in the demineralized hydroxyapatite. Calcium ions are re-embedded into the apatite lattice [17] and the resulting fluoridated hydroxyapatite is less soluble, in the event of an acidic attack. In vitro enamel remineralization experiments have demonstrated that with increasing concentrations of fluoride, the calcium loss from enamel was reversed under acidic conditions [18]. Results of the present study showed that at the micrometer level, prevention of demineralization was concentration dependent. 5000 ppm fluoride decreased mineral loss and lesion depth significantly as compared to 250 ppm fluoride.

Clinical short-term[19,20], and long-term studies [21-23] have shown that the combination of organic amine fluoride with stannous fluoride (ASF) is an effective antibacterial and consequently, a plaque inhibiting agent. ASF inhibited both the metabolic activity of different oral bacteria as well as the acid production [24,25]. In our study, the ASF treated group, however, showed no prevention of demineralization. On a clinical level, the type of fluoride i.e. ASF or NaF, and the form of application i.e. dentifrice or mouthrinse, have been found to have the same effect on the development of new root caries lesions [26].

In the present study, 5000 ppm NaF was more effective in preventing mineral loss and lesion depth than 250 ppm ASF. However, a direct comparison between the NaF and ASF was not the aim of this study. The higher concentration of fluoride (NaF) was more efficient in terms of lesion depth and mineral loss reduction when compared to ASF and to water.

Root caries can be successfully treated non-invasively with a fluoride toothpaste. A 5000 ppm fluoride toothpaste was significantly more effective in remineralizing primary root caries lesion than toothpaste containing 1100 ppm F [5,27] The data of the present study provides evidence to support these results.

As compared to chemical analysis [28], TMR is a sensitive and valid method for detecting loss of ions and has the further advantage of providing information on the topography of the mineral volume percentage distribution in tooth tissue. The TMR methodology was implemented in our study for the determination of the change of mineral content during demineralization.

In order to control variables between different volunteers, it was decided to include only one volunteer in the study. At the same time, an adequate number of specimens (n = 45) were used to show that the differences were valid. The focus of this study was intended to be only on the effects of fluoride on the dentin surfaces, with the effect of the fluoride being the sole variable factor. Caries is a complex phenomenon influenced by systemic defense factors, such as salivary flow and buffering capacity, salivary and microbial interactions, adhesion and co-adhesion of microorganisms and a number of external factors – for example, diet, oral hygiene, and fluoride availability. There is no doubt that the above-mentioned factors play a significant role in dentin demineralization. Consequently, the results of this study need be confirmed with another study involving more volunteers.

In light of the above results, a further in situ and a clinical study designed to investigate the effects of 5000 ppm fluoride on the demineralization of dentin surfaces is planned.

## Conclusion

On the basis of the results and within the limitations of this study, we conclude that the use of a 5000 ppm fluoride treatment may be beneficial in preventing dentin demineralization. However, further studies are necessary to evaluate the clinical behaviour of the application of 5000 ppm fluoride on dentin surfaces.

## List of abbreviations

ASF: amine fluoride and stannous fluoride; NaF: sodium fluoride; F: Fluoride; ppm: parts per million; TMR: transversal microradiography.

## Competing interests

The authors declare that they have no competing interests.

## Authors' contributions

MB responsible for, conception and design of the study, acquisition of data, analysis and interpretation of data and writing the manuscript. Y-H P C helped to draft the manuscript. M-T W carried out the study. M-J A carried out the transversal microradiography. W H-M R conception and design of the study. SZ participated in its design and helped to draft the manuscript. All authors have read and approved the final manuscript.
